# RNA-seq identifies Amd1 as a regulator of hepatocyte proliferation via Skp2 during liver development and hepatocellular carcinoma progression in zebrafish

**DOI:** 10.1016/j.gendis.2024.101486

**Published:** 2024-12-11

**Authors:** Ke Zhang, Botong Li, Zhiling Deng, Yong Dong, Yuanyuan Li, Bingyu Chen, Mao Lu, Liyan Wang, Xingdong Liu, Zhenhua Guo, Sizhou Huang

**Affiliations:** a Development and Regeneration Key Laboratory of Sichuan Province, Department of Anatomy and Histology and Embryology, School of Basic Medical Sciences, Chengdu Medical College, Chengdu, Sichuan 610500, China; b Department of Neurology, The Second Affiliated Hospital of Chengdu Medical College, China National Nuclear Corporation 416 Hospital, Chengdu, Sichuan 610000, China; c Department of Dermatovenereology, The First Affiliated Hospital of Chengdu Medical College, Chengdu, Sichuan 610500, China; d Ministry of Education Key Laboratory of Child Development and Disorders, Key Laboratory of Pediatrics in Chongqing, CSTC2009CA5002, Chongqing International Science and Technology Cooperation Center for Child Development and Disorders, Children's Hospital of Chongqing Medical University, Chongqing 400014, China; e Central Hospital of Suining, Suining, Sichuan 629000, China

Early studies utilized microarrays to identify liver-enriched genes, and the roles of some of these genes in liver development in mice and zebrafish were confirmed.^1^^,^^2^ However, many genes involved in liver growth regulation remained unidentified through this approach. Recently, bulk RNA sequencing or single-cell RNA sequencing has been used to study liver differentiation,^3^ but no study has screened genes highly expressed in hepatocytes during liver growth. Since rapid hepatocyte proliferation is a common characteristic of liver growth in early liver development and hepatocellular carcinoma (HCC) progression, identifying the genes being involved in regulating liver growth and clarifying how it works would benefit studying HCC progress.

We observed that the zebrafish liver underwent rapid growth between 2 dpf (days post fertilization) and 5 dpf (Fig. 1A; Fig. S1) and hypothesized that certain genes regulating liver growth were likely to be highly expressed in hepatocytes during this period. To identify these genes, we performed RNA sequencing on GFP-labeled hepatocytes sorted at different developmental stages (Fig. 1B): 3 dpf, 7 dpf and adult zebrafish. The results showed that 1654 genes were enriched in the hepatocytes but not in non-hepatocytes at 3 dpf (Table S1). Among the top 25 of these genes, 14 were previously reported as liver-enriched in the Zfin database, 2 had been identified in earlier publications, and 9 were novel findings (Fig. 1C; Table S3), suggesting the identification of new liver-enriched genes. Further analysis revealed that 813 of these genes were highly expressed in 3 dpf hepatocytes but not in adult hepatocytes (Table S4). This finding was validated by quantitative reverse transcription PCR analysis of four selected genes (Fig. 1D). Since the rate of liver growth significantly decreased after 5 hpf (Fig. 1A; Fig. S1), we hypothesized that the expression of genes regulating liver growth would be down-regulated in hepatocytes at 7 dpf compared with 3 dpf. Indeed, 756 of the 813 genes were down-regulated in hepatocytes at 7 dpf compared with 3 dpf (Table. S5). Interestingly, among these 756 genes, four were associated with arginine and proline metabolism, and quantitative reverse transcription PCR confirmed their higher expression in 3 dpf hepatocytes (Fig. 1E). To these four genes, *amd1*, the key enzyme involved in the synthesis of polyamines and embryonic stem cell self-renewal,^4^ its role in liver development has not been addressed. We investigated the role of *amd1* in liver growth to confirm that the genes we identified could serve as candidates for regulating liver growth.

**Figure 1:**
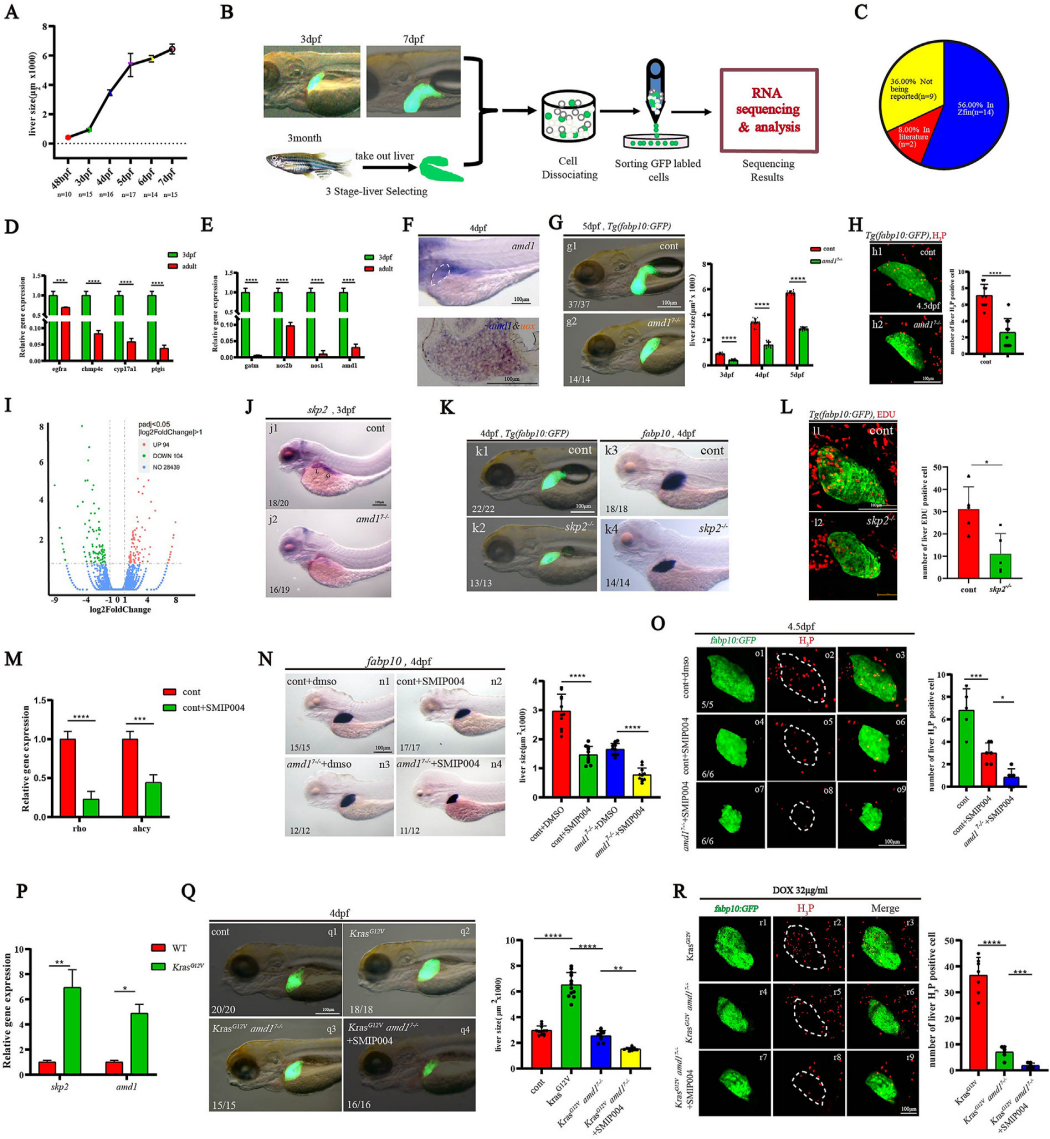
The *amd1*-*skp2* cascade regulates hepatocyte proliferation during liver development and HCC progression in zebrafish. **(A)** Comparison of liver size from 2 dpf to 7 dpf using *in situ* hybridization and live *Tg(fabp10:GFP)* transgenic embryos. Lateral view. **(B)** Schedule for sorting different staged hepatocytes and bulk RNA sequencing. **(C)** Among the top 25 genes of liver-enriched genes at 3 dpf, 56% were reported to be enriched in the embryonic liver in the Zfin database, 8% were reported in early literature, and 36% were uncharacterized. **(D)** Four genes were randomly selected from those enriched and highly expressed in hepatocytes at 3 dpf. Their expression levels were higher in hepatocytes at 3 dpf compared with adult hepatocytes. **(E)** The relative expression of *gatm* (0.63%), *nos2b* (9.7%), *nos1* (1.0%), and *amd1* (3.0%) in 3 dpf hepatocytes was much higher than in adult hepatocytes. **(F)** Single staining (*amd1* as probe) and double staining (*uox* for fast red, *amd1* for blue) at 4 dpf. *Uox* and *amd1* were colocalized in the liver. **(G)** From 2 dpf to 5 dpf, liver size was smaller in *amd1*^*7−/−*^ embryos than in controls. **(H)** At 4.5 dpf, the number of proliferating hepatocytes stained with H3P in *amd1*^*7−/−*^ embryos was lower than in control embryos. **(I)** RNA sequencing data analysis showed that in *amd1*^*7−/−*^ embryos, 94 genes were up-regulated, and 104 genes were down-regulated; there was no significant difference in the expression of 28,439 genes. **(J)***In situ* experiments showed that *skp2* was down-regulated in the liver and gut in *amd1*^*7−/−*^ embryos at 4 dpf. **(K)** Liver size in *skp2* mutants (k2, k4) was smaller than in controls (k1, k3). **(L)** The number of proliferating hepatocytes stained with EdU in *skp2* mutants (l2) is smaller than in controls (l1). **(M)** Quantitative reverse transcription PCR results showed that the expression of *rho* and *ahcy* was significantly down-regulated in embryos treated with 80 μM SMIP004. **(N)** In situ experiments showed that 100% of embryos treated with 80 μM SMIP004 (Nn2) displayed smaller livers than controls (Nn1). Inhibiting skp2 activity using SMIP004 resulted in 91.6% of amd1^*7−/−*^ embryos (Nn4) displaying much smaller livers than controls (Nn3). **(O)** Cell proliferation was evaluated using H_3_P staining. After treatment with SMIP004, the number of H_3_P-stained hepatocytes was decreased compared with controls (control, *n* = 5; SMIP treatment, *n* = 6; *p* = 0.0004). SMIP004 treatment further decreased the number of H_3_P-stained hepatocytes in amd1^*7−/−*^ embryos (*n* = 6; *p* = 0.171). **(P)** Compared with normal liver, the expression of *amd1* (5.39-fold increase; *p* = 0.0162) and *skp2* (7.93-fold increase; *p* = 0.0035) was significantly increased. **(Q)** Dox-induced overexpression of *Kras* increased liver growth in the HCC model (Qq2; *p* ≤ 0.0001), *amd1* loss of function decreased the size of the liver in the HCC model (Qq3; *p* ≤ 0.0001), and simultaneous inhibition of *skp2* and *amd1* resulted in much smaller livers in the HCC model (Qq4; *p* = 0.0012). **(R)** Compared with controls, *amd1* loss of function decreased the number of H_3_P-stained hepatocytes (Rr5; *p* ≤ 0.0001), and simultaneous inhibition of *skp2* and *amd1* further decreased the number of H_3_P-stained hepatocytes (Rr8; *p* = 0.0005). Values are reported as mean ± standard error of the mean. ∗*p* < 0.05, ∗∗*p* < 0.01, ∗∗∗*p* < 0.001, ∗∗∗∗*p* < 0.0001. HCC, hepatocellular carcinoma; dpf, days post fertilization.

First, *in situ* experiments showed that *amd1* was a maternal gene and was highly expressed in the liver at 3 dpf and 4 dpf (Fig. 1F; Fig. S2A, B). The expression of *amd1* in hepatocytes at 3 dpf was higher than at 4 dpf (Fig. S2C), coinciding with the most rapid liver growth occurring between 3 dpf and 4 dpf (Fig. 1A; Fig. S1). These data suggest a potential role for *amd1* in liver growth. Next, we generated two *amd1* mutant lines (*amd1*^*168*^ and *amd1*^*7*^; Fig. S3A–D) to investigate this possibility. Although no distinct external phenotype was observed during early development in *amd1*^*7−/−*^ or *amd1*^*168−/−*^ embryos (Fig. S3E, F), and liver specification was not disturbed (Fig. S4), the livers in these two mutant lines were smaller than those of controls from 3 dpf to 5 dpf (Fig. 1G; Fig. S5). These results indicate that *amd1* mutation specifically inhibits liver growth. Furthermore, immunostaining and TUNEL assays showed that hepatocyte proliferation, but not apoptosis, was decreased in *amd1*^*7−/−*^ embryos (Fig. 1H; Fig. S6B–E). Additionally, proliferation-related markers *cdk1*, *cdk4*, *chk1* and *mcm5* were down-regulated in *amd1* mutants (Fig. S6A). These findings demonstrate that *amd1* is essential for hepatocyte proliferation.

To elucidate how *amd1* regulates liver growth, we analyzed gene expression in *amd1*^*7−/−*^ embryos at 4 dpf using RNA sequencing. The results showed that 94 genes were up-regulated and 104 genes were down-regulated in *amd1*^*7−/−*^ embryos (Fig. 1I). Among the down-regulated genes, four were in the mTOR signaling pathway, including *skp2* (Fig. S7A, B). *Skp2*, a key component of the SKP1-cullin 1-F-box (SCF) complex, primarily functions as an oncoprotein.^5^ *Skp2* was expressed ubiquitously before 24 hpf and then restricted to the liver, gut, eyes and boundary of the hindbrain and midbrain at 3 dpf (Fig. S7C). Quantitative reverse transcription PCR (Fig. S7D) and *in situ* hybridization (Fig. 1J) showed that *skp2* was down-regulated in *amd1*^*7−/−*^ embryos at 3 dpf, particularly in the liver and gut. These results suggest that *skp2* may be required for *amd1* to regulate liver growth. Next, we generated a *skp2* mutant line (Fig. S8A–D) and found that both liver size and hepatocyte proliferation were reduced (Fig. 1K and L). Finally, we found that injection of *skp2* mRNA could restore liver development in *amd1* mutants (Fig. S8E). These data suggest that *skp2* is required for liver development and is also essential for *amd1* to regulate liver development.

*Skp2* was ubiquitously expressed at early stages (Fig. S7C), and the expression of *prox1* and *hhex* was decreased in *skp2* mutants at 48 hpf (Fig. S8H, I). This result suggests the possibility that the liver phenotype is a secondary effect of early developmental defects in *skp2* mutants. Treatment with 80 μM SMIP004 (*skp2* inhibitor) reduced *Skp2* activity, leading to decreased expression of the downstream genes *rho* and *ahcy* (Fig. 1M; Fig. S9A–D). To observe the direct role of *skp2* during liver growth, embryos were treated with 80 μM SMIP004 from 48 hpf to 4 dpf, and liver size was analyzed (Fig. S9E, F). The data showed that *skp2* inhibition resulted in smaller liver at 4 dpf (Fig. 1Nn2; Fig. S9Ff2) and decreased hepatocyte proliferation (Fig. 1Oo6), demonstrating that *skp2* plays a vital role in liver growth. Additionally, treatment with SMIP004 also led to a much smaller liver and significantly reduced hepatocyte proliferation in *amd1*^*7−/−*^ embryos (Fig. 1Nn4, Oo9), implying that *skp2* is required for *amd1* to regulate liver growth.

Since hepatocytes in developing liver and HCC are characterized by rapid cell proliferation, we generated an HCC model in zebrafish larvae (Fig. S10A–C) to examine whether *amd1* and *skp2* played critical roles during zebrafish HCC progression. The data showed that the expression of *amd1* and *skp2* was up-regulated in the sorted HCC cells (Fig. 1P), and the liver size and hepatocyte proliferation in *amd1*^*−/−*^ embryos were decreased compared with controls (Fig. 1Qq3, Rr6). Additionally, simultaneous inhibition of *amd1* and *skp2* further decreased liver size and hepatocyte proliferation in the zebrafish HCC model (Fig. 1Qq4, Rr9). These data demonstrate that the *Amd1*-*Skp2* cascade was also involved in HCC progression in zebrafish.

In conclusion, our study identified some uncharacterized genes enriched in hepatocytes at 3 dpf. Among these genes, *amd1* was identified as playing a crucial role in regulating hepatocyte proliferation via *skp2* during normal liver development and HCC progression. These results imply that the identified genes may be candidates for regulating hepatocyte proliferation in normal liver development and HCC progression.

## CRediT authorship contribution statement

**Ke Zhang:** Validation, Software, Methodology, Investigation, Formal analysis, Data curation. **Botong Li:** Methodology, Investigation, Data curation. **Zhiling Deng:** Methodology, Investigation. **Yong Dong:** Software, Methodology, Investigation. **Yuanyuan Li:** Methodology, Investigation. **Bingyu Chen:** Investigation. **Mao Lu:** Writing – review & editing, Methodology. **Liyan Wang:** Writing – review & editing, Methodology. **Xingdong Liu:** Writing – review & editing, Conceptualization. **Zhenhua Guo:** Writing – review & editing, Writing – original draft, Visualization, Validation, Resources, Methodology, Investigation, Formal analysis, Conceptualization. **Sizhou Huang:** Writing – review & editing, Writing – original draft, Supervision, Methodology, Investigation, Funding acquisition, Conceptualization.

## Ethics declaration

All experimental methods and protocols were approved by Chengdu Medical College (Sichuan, China). Zebrafish were maintained following the Guidelines of the Animal Care Committee of Chengdu Medical College, Sichuan, China.

## Funding

This work was supported by the 10.13039/501100001809National Natural Science Foundation of China (No. 32070805), the 10.13039/501100004829Science and Technology Department of Sichuan Province, China (No. 2024NSFSC2093); National Clinical Research Center for Child Health and Disorders, Children’s Hospital of Chongqing Medical University (NCRCCHD-2022-GP-04).

## Conflict of interests

The authors declared no conflict of interests.
