# Supplementary material for: RNA-seq identifies Amd1 as a regulator of hepatocyte proliferation via Skp2 during liver development and hepatocellular carcinoma progression in zebrafish
Source: Genes Dis. 2024 Dec 11;12(5):101486. doi: 10.1016/j.gendis.2024.101486 (PMC12142500; doi:10.1016/j.gendis.2024.101486)
Supplement: Multimedia component 1 [file mmc1.docx]

**Supplemental Material**

**Materials and Methods**

**Fish and Fish Maintenance**

Wild-type (AB), transgenic line *Tg(fabp10:RTTA),Tg(Tetre:EGFP-kras_G12V)* ^1^*, Tg(fabp10:EGFP)* ^2^, *skp2^+/-^* and *amd1^+/-^* line fish were maintained in standard conditions at approximately 28.5 °C. The developmental stages were characterized as previously described ^3^.

***Amd1* and *skp2* mutant construction**

One sgRNA was designed according to the exon 1 region of the *amd1* gene, and the sgRNA was synthesized in vitro as described in the manual ([HiScribe™ T7 High Yield RNA Synthesis Kit，](https://international.neb.com/products/e2040-hiscribe-t7-high-yield-rna-synthesis-kit)NEB, NO. E2040S), the sgRNA and Cas9 protein were mixed and coinjected into the cell cytoplasm at the one-cell stage. Several injected embryos were used to evaluate the mutation efficiency using sequencing, and the remaining embryos were designated F0 (Founder). F0 adult fish was crossed with wild type to obtain F1 embryos. After evaluating the mutation efficiency using part of F1 embryos, the remaining F1 was maintained and grown to produce F2 embryos. Two types of useful mutations were identified in F2. The targeted sequence of *amd1* sgRNA1 was 5’- GGAGGTGTGGTTCTCCCGGC-3’. The primers used for amplifying the targeted genomic DNA (used for sequencing) were *amd1*-check*-*F: 5′- GTGATTCCATCCGACGGTTTA -3′ and*amd1*-check*-*R: 5′- CTGACATTATCACAGCGTTTCAC -3′. To obtain mosaic mutants for *skp2* in F0 embryos, we optimized the CRISPR-cas9 gene editing process according to a previous report ^38^. In brief, three sgRNAs for *skp2* were designed and synthesized in vitro using [HiScribe™ T7 High Yield RNA Synthesis Kit](https://international.neb.com/products/e2040-hiscribe-t7-high-yield-rna-synthesis-kit)(NEB, E2040S). The Cas9-sgRNA ribonucleoprotein complex (RNP) solution was prepared as follows: Cas9 protein (EnGen^®^ Spy Cas9 NLS, NEB, M0646T) 1.3μl, 1 M KCl 1.1μl, total sgRNA 2500 ng (830ng each), phenol red 0.3μl. Finally, RNA-free H_2_O was added to 5μl total, mixed completely, incubated at 37 °C for 5 minutes, and then placed back into an ice bath for the following injection procedure. The RNPs were injected into the embryo yolk within 15 minutes after fertilization. To examine the mutant efficiency for *skp2*, 16 injected embryos were used to prepare genomic DNA, and semi-quantitative RT‒PCR was performed for evaluation as described in a previous report ^6^. The primers used are shown in Table S1. To get the stable *skp2* mutant lines, the method being used to screen *amd1* mutant was used to screen *skp2* mutant. In our work a frame shift mutant line with a premature stop codon was obtained.

**Bulk RNA Sequencing**

To compare the transcriptome of hepatocytes in different liver stages, GFP-labelled hepatocytes were sorted using flowcytometry (Moflflo XDP, Beckman) from transgenic line *Tg(fabp10:GFP)* embryos and adult livers. Approximately 400 hepatocytes were collected for each stage. cDNA libraries were generated from these sorted cells using the Smart-seq2 protocol. RNA sequencing was performed using the PE100 strategy (HiSeq 2500, Illumina). Sequencing data were analysed as previously reported ^4^. To compare the transcriptome of wild-type and *amd1^7-/-^* embryos at 4 dpf, total RNA was prepared using TRIzol according tothe manual. RNA sequencing and analysis were performed by Novogene Co., Ltd. (Tian Jin, China).

**Fin-clip and identifying *amd1* mutation embryos**

Since there is no clear morphological phenotype for *amd1^-/-^* embryos, to identify *amd1* homozygotes, the tail fin of zebrafish larvae was cut at 48 hpf, and genomic DNA was individually prepared as following: After anaesthetizing the embryo, the tip of the tail was cut with a scalpel to prepare genomic DNA as previously reported ^5^, and the embryos were kept for further experiments. Genomic DNA was used to amplify the target region using PCR. Then, we evaluated whether the larvae were homozygotes according to the PCR results. For the wild-type larvae and heterozygotes, the target fragment was obtained, while for the homozygotes, the target fragment was not amplified, no amplicon was detected. The primers used here were as follows: *amd1*-screen-F: 5’- GTGGTTCTCCCGGCAG-3’, *amd1*-screen*-*R: 5′- CTGACATTATCACAGCGTTTCAC -3′.

**Chemical treatment**

SMIP004 was used to inhibit the function of *skp2* as described in a previous report ^7^. Two concentrations, 40 μM and 80 μM, were selected to inhibit *skp2* activity. The chemical SMIP004 was diluted with egg water to the concentration described above. The embryos were incubated with SMIP004 solution (40 μM or 80 μM) from 3 hpf to 24 hpf to evaluate which concentration was the proper concentration. Then, the embryos were incubated with SMIP004 solution (proper concentration: 80 μM) from 48 hpf to the stages needed.

**Plasmid Construction**

Total RNA was extracted following the manufacturer’s instructions (TRIzol, Ambion, 15596-026). cDNA was prepared using a Revert Aid First Strand cDNA Synthesis Kit (Fermentas, K1622) according to the manufacturer’s instructions. The CDs of *amd1* and *skp2* were amplified individually using PCR (Prim STAR Max Premix Takara, R045A) and cloned into the PCS^2+^ vector (5x In-Fusion HD Enzemy Premix, Takara, 639649). The primers for cloning were as follows: PCS^2+^_F: 5′-CTCGAGCCTCTAGAACTATAGTG-3′, PCS^2+^_R: 5′-TGGTGTTTTCAAAGCAACGATATCG-3′, *amd1-pcs2+_*F: 5′-TCTTTTTGCAGGATCGGAGTCTGTTTGTCTCACGATGG-3′, *amd1-pcs2+_*R: 5′-GTTCTAGAGGCTCGACGCTTCTTCATGTCAGAGGATCAG-3′, *skp2-pcs2+*F:5′-GCTTTGAAAACACCACAAGTCAGGATGTCAAACGAAAGG-3′, *skp2-pcs2+*_R: 5′-GTTCTAGAGGCTCGAGCATTAATGTTTGTAGACGAGTCTGC-3′.

**mRNA injection**

*skp2* mRNA was synthesized in vitro using an mMESSAGE Kit (Ambion, AM1340) as the described in the manual. The concentration for *skp2* mRNA injection was 40ng/μl. *skp2* mRNA was injected at the 1-4 cell stage.

**RT‒qPCR**

RT‒qPCR was performed using the Brilliant III Ultra-Fast SYBR Green QPCR Master Mix (Agilent Technologies) and the CFX96 Real-Time System (BIO-RAD) according to the manufacturer’s instructions. The amount of *beta-actin* was used for normalizer. The primers are listed in Table S1. All experiments were repeated at least 3 times.

**Whole-mount *in situ* hybridization and section**

One color *in situ* hybridization was performed as described in a previous study ^8^. The previous probes *fabp10, prox1, hhex,* and *fabp2* were used as described in previous reports ^9^. The CDs of *amd1* and *skp2* were amplified using PCR and cloned into the vector pcs2^+^, then linearized the plasmids and synthesized the individual antisense probe as previously reported ^9^. Two color *in situ* hybridization was performed as described in a previous study ^10^. Specifically, digoxygenin-labeled *amd1* probe and fluorescein - labeled *uox* probe were used in our study. For sectioning, in situ hybridized embryos were re-fixed in 4% paraformaldehyde in PBS, followed by incubation in 15 and 30% sucrose in 0.1% Tween/phosphate buffered saline for 2 hours each. Then, embryos were mounted in 1.5% agarose in 30% sucrose, and balanced in 30% sucrose solution overnight at 4 °C. The mounted embryo was re-mounted in OCT (Sakura), sectioned using a CM1850 cryostat (Leica),

**Immunostaining**

The embryos were fixed overnight with PFA (4% in PBS) at 4 °C, washed with PBS (5 min, 3x) and blocked with PBTN (4% BSA, 0.02%NaN_3,_ in PT) for 2 hours at 4 °C. Then, the primary antibody against H3p (GTX128116) or Caspase3 (BD 559565) was diluted with PBTN at 1:200 and incubated on a shaker at 4 °C overnight. Then, the embryos were washed with PT (0.3% Triton-X-100, in 1X PBS) for at least 20 min 8 times. The secondary antibody, Donkey anti rabit IgG, Texas Red coupled; GeneTex 26800) or Alexa FluorTM 647（invitrogen, A21244） was diluted with PBTN in 1:500 and added. The embryos were incubated overnight at 4 °C (kept in the dark). Finally, the embryos were washed with PT more than 8 times (30 min each time) and imaged.

**EDU experiment**

BeyoClick™ EdU Cell Proliferation Kit with Alexa Fluor 594 (Beyotime, C0078S) was used for this experiment. 200uM EDU solution containing 2%DMSO and 0.01% phenol red was prepared. Zebrafish embryos were mounted with low melting point agar (0.8%) and the EDU solution was injected pericardially. After injection, the embryos were replaced in egg water at 28.5℃ for 40 minutes, then fixed them overnight at 4℃ with PEM. The fixed embryos were rinsed with PBS for 3 times (5 min each time) and treated with pre-cooled acetone at -20℃ for 40 min, then were washed by PT for 3 times (20 minutes each time). Next the embryos were incubated with 3%BSA at room temperature for 2 hours and washed with PT 3 times, then according the manual the EDU reaction solution was added and keep the reaction for 30 minutes in dark. After EdU staining reaction the embryos were washed with PT for 3 times, flowing Immunostaining for GFP.

**TUNEL** **staining**

Embryos were fixed in 4% PFA overnight at 4 °C, washed with PBST 3 times (10 minutes each time) and stored in 100% methanol overnight. Then, the embryos were washed 3 times with PBST 3 times, and an In Situ Cell Death Detection Fluorescein kit (Roche11684795910) was applied to examine cell apoptosis according to the manufacturer’s instructions.

**Microscopy**

Images of whole-mount *in situ* hybridized embryos (mounted in 80%-100% glycerol) and section samples were captured at room temperature using an OLYMPUS SZX16. To examine positive pro-apoptotic, proliferating cells in the liver, the *Tg(fabp10:EGFP)* embryos were fixed in PFA (4% in PBS) overnight and mounted in 1.5% Low Melting-point Agar. Then, the proliferating cells and apoptotic cells were captured at room temperature using an OLYMPUS FLUOVIEW FV1000.

**Statistical analysis**

In this work, the liver size was compared using the area of liver with lateral view. The proliferating hepatocytes (GFP) labelled with EdU or H3P in the 3D liver image (the overlapped image with all z-stick single images. Generally, five images were captured for each liver.) was compared between controls and mutants. The data were analysed with Novoexpress, ImageJ, statistical software in GraphPad Prism 8 for Windows (GraphPad Software). Quantitative data are presented as the means S.D. Experiments were performed at least three times for each experiment. NS, not significant, “*” p < 0.05, “**” p < 0.01, “***” p < 0.001 and “****” p < 0.0001.

**References**

1. Nguyen, A.T., et al., Development of a conditional liver tumor model by mifepristone-inducible Cre recombination to control oncogenic kras V12 expression in transgenic zebrafish. *Sci Rep*, 2016. **6**: p. 19559.

2. Zhu, C., et al., Aplnra/b Sequentially Regulate Organ Left-Right Patterning via Distinct Mechanisms. *Int J Biol Sci*, 2019. **15**(6): p. 1225-1239.

3. Kimmel, C.B., et al., Stages of embryonic development of the zebrafish. *Dev Dyn*, 1995. **203**(3): p. 253-310.

4. Liu, C., et al., Macrophages Mediate the Repair of Brain Vascular Rupture through Direct Physical Adhesion and Mechanical Traction. *Immunity*, 2016. **44**(5): p. 1162-76.

5. Wilkinson, R.N., et al., A method for high-throughput PCR-based genotyping of larval zebrafish tail biopsies. *Biotechniques*, 2013. **55**(6): p. 314-6.

6. Wu, R.S., et al., A Rapid Method for Directed Gene Knockout for Screening in G0 Zebrafish. *Dev Cell*, 2018. **46**(1): p. 112-125 e4.

7. Li, C., et al., SKP2 promotes breast cancer tumorigenesis and radiation tolerance through PDCD4 ubiquitination. *J Exp Clin Cancer Res*, 2019. **38**(1): p. 76.

8. Liu, J., et al., Chemokine signaling links cell-cycle progression and cilia formation for left-right symmetry breaking. *PLoS Biol*, 2019. **17**(8): p. e3000203.

9. Zhang, Y., et al., Mcm5 Represses Endodermal Migration through Cxcr4a-itgb1b Cascade Instead of Cell Cycle Control. *Biomolecules*, 2022. **12**(2).

10. Dunn, K., A. Vashisht, and D.R. Hammond-Weinberger, Comparative in situ hybridization protocols in zebrafish. *Biotechniques*, 2022. **73**(3): p. 123-130.

**Figure S1-S10**

**Supporting information**


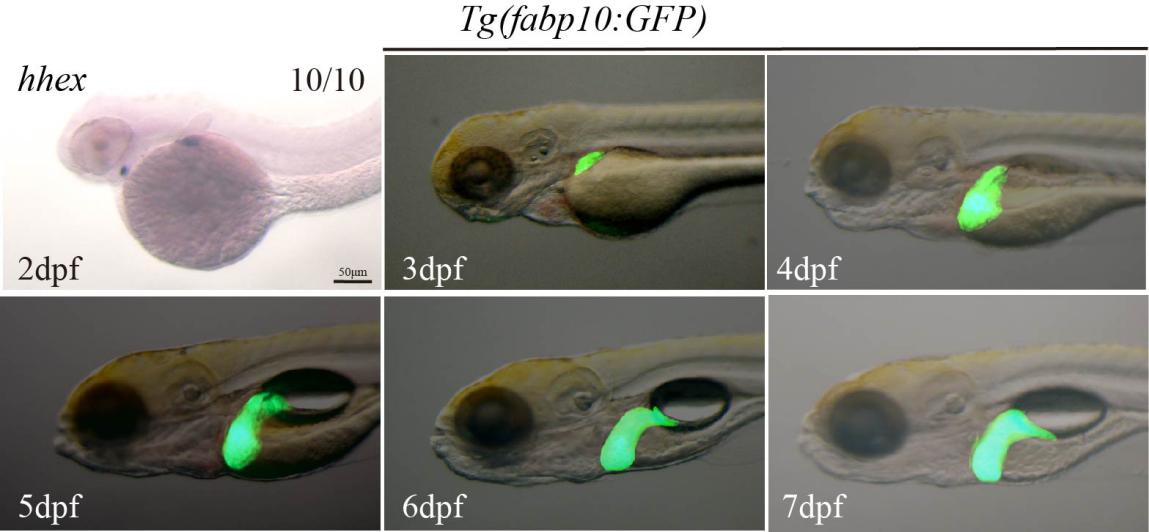


**Figure S1. liver growth**

Liver size comparing from 2dpf to 7dpf using *in situ* hybridization and living *Tg(fabp10:GFP)* transgenic embryos. Lateral view.


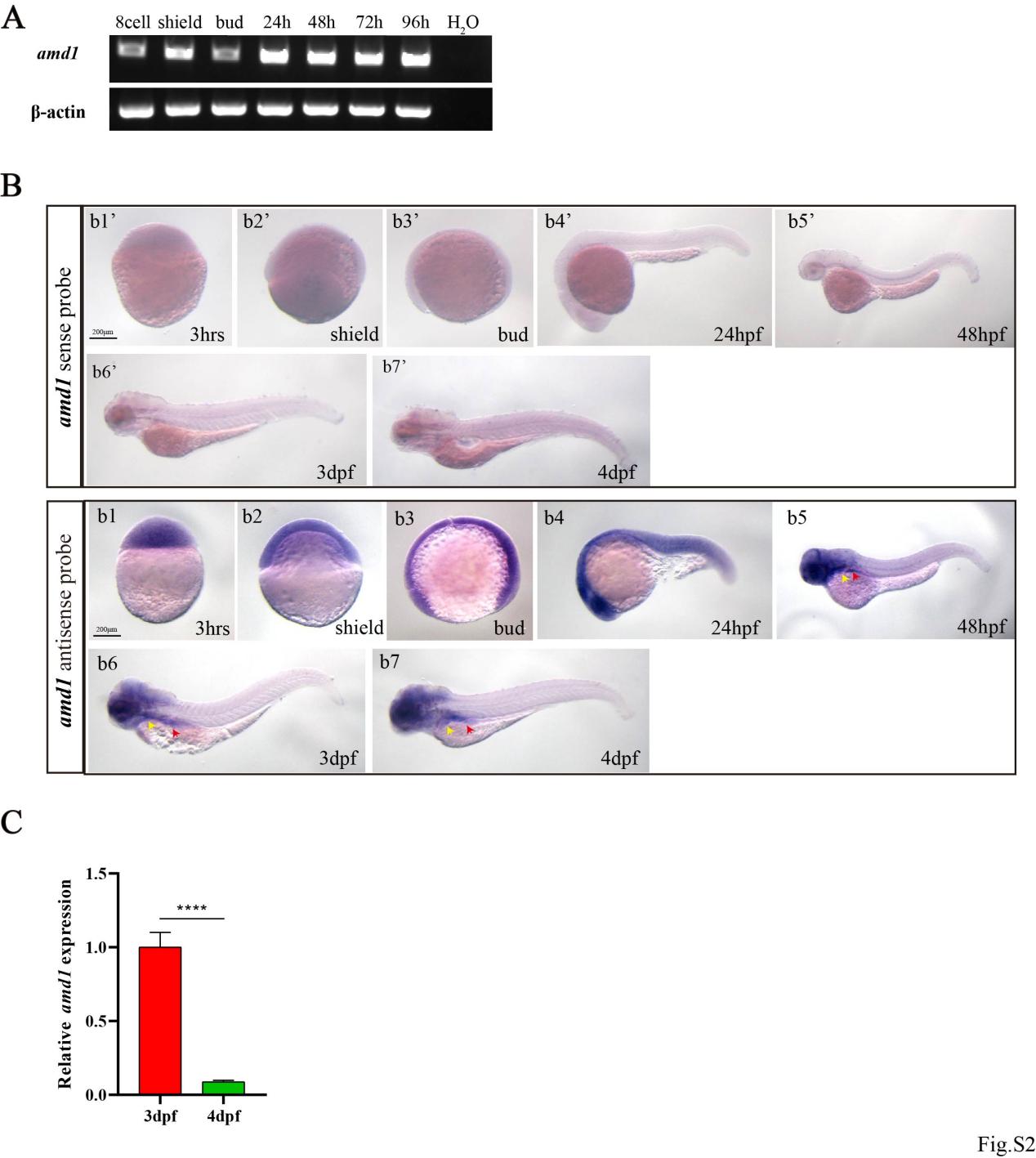


**Figure S2. The expression pattern of *amd1* in early embryonic development**

(A, B) The expression of *amd1* in early embryonic development. (A) PCR amplification for *amd1* in embryos at 8-cell stage, shield stage, bud stage, 24hpf, 48hpf, 72hpf and 96hpf. (B) *In situ* hybridization staining for *amd1* sense probe (Bb1’-b7’) and antisense probe (Bb1-b7). Especially from 2dpf to 4dpf, *amd1* was expressed highly in endoderm cells (Bb5-b7, red arrow showed), including in liver (Bb6-b7).The expression level of *amd1* in hepatocytes on 4dpf is 8.9% of that in hepatocytes on 3dpf （C）. Values are reported as mean ± SEM. “****” P < 0.0001. Scale bars, 200μm.


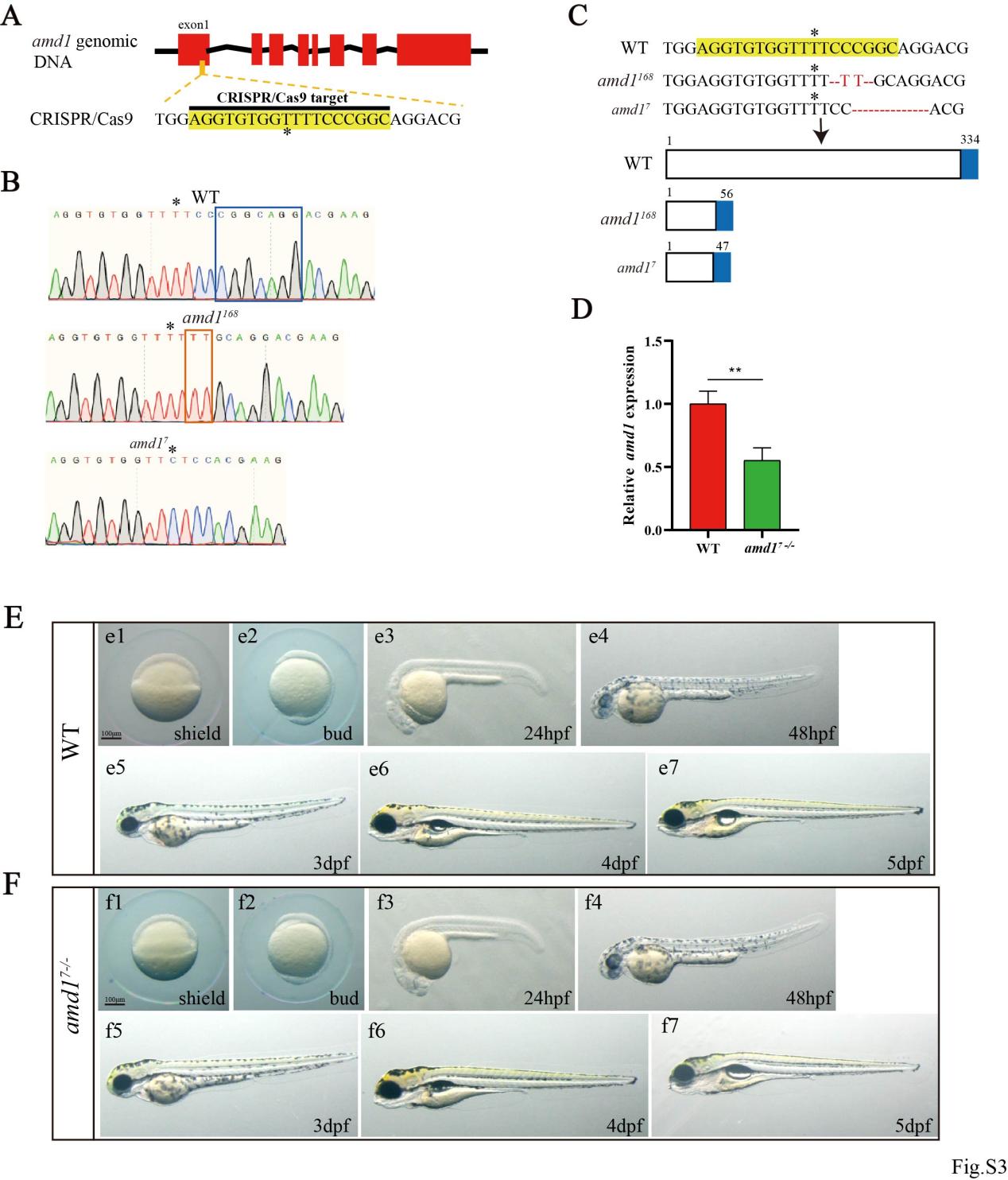


**Figure S3. *Amd1* mutant generation and the embryonic appearance of living wild type embryos and *amd1* mutants**

(A) Target of *amd1* sgRNA in exon1 of *amd1* gene. (B) The sequencing result of amd1 wild type, *amd1^168-/-^*and *amd1^7-/-^* , the nucleotide labeled with “*” is single nucleotide polymorphism (SNP), here the nucleotide can be “T” or “C” in *amd1* wild type embryos. (C) The nucleotide sequence highlighted with yellow is the target sequence. In *amd1* mutant1 (*amd1^168-/-^*) the sequence “CCCG” was changed to “TT”. In *amd1* mutant2 (*amd1^7-/-^*) the sequence “CGGCAGG” was deleted. Both mutations led to frame-shift and a premature stop codon. (D) The expression of *amd1* in *amd1^7-/-^* hepatocytes is 55.2% of that in wild type hepatocytes on 4dpf. Values are reported as mean ± SEM. “**” P < 0.01. (Ee1-e7) The embryonic appearance of living wild type embryos at shield stage (Ee1), bud (Ee2), 24hpf (Ee3), 48hpf (Ee4), 3dpf (Ee5), 4dpf (Ee6) and 5dpf (Ee7). (Ff1-f7) The embryonic appearance of living *amd1^7-/-^* embryos at shield stage (Ff1), bud (Ff2), 24hpf (Ff3), 48hpf (Ff 4), 3dpf (Ff 5), 4dpf (Ff 6) and 5dpf (Ff 7). No distinct difference exists between wild type embryos and *amd1^7-/-^* embryos at different stages. Scale bars, 100μm.


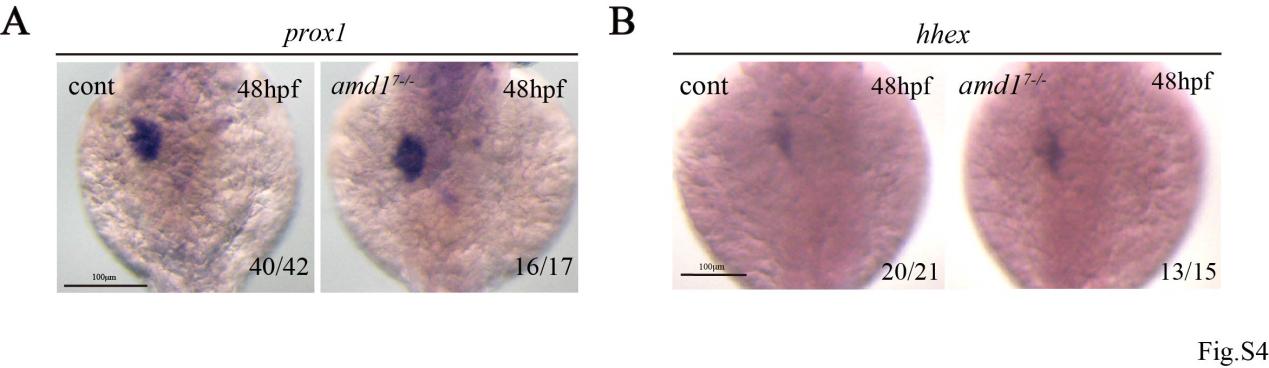


**Figure S4.Expression of *prox1* and *hhex*in control embryos and *amd1^7-/-^* embryos at 48hpf**

(A, B) Expression of early liver marker *prox1* and *hhex* in embryos at 48hpf. There was no distinct difference for the expression of *prox1* (A, n=17) and *hhex* (B, n=15) between controls and *amd1^7-/-^* embryos at 48hpf. Scale bars, 100μm.


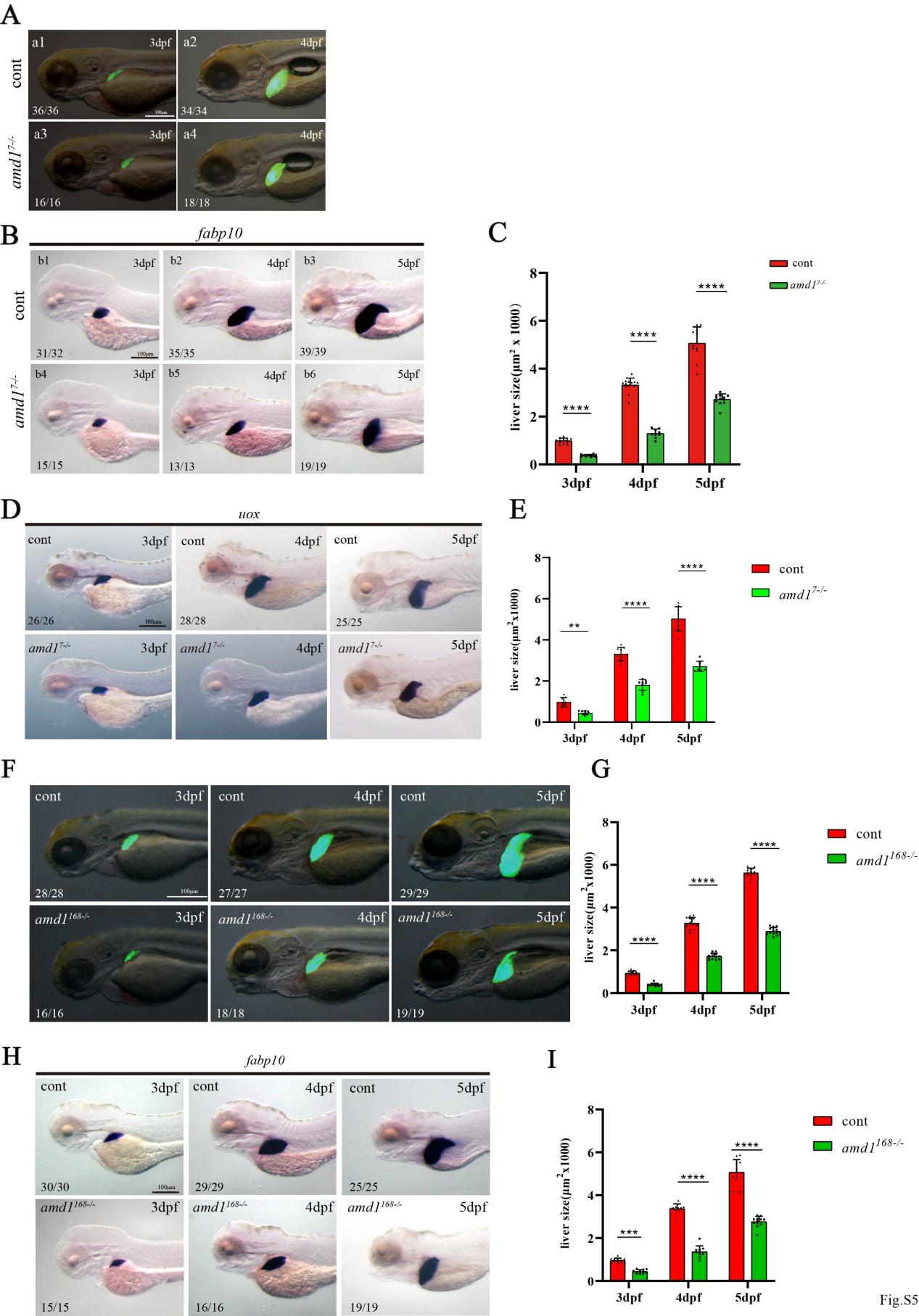


**Figure S5. The liver developmental defect in *amd1^-/-^* embryos**

(A) Comparing the liver size in *amd1^7-/-^* embryos and control embryos using *Tg(fabp10:GFP)* transgenic line. From 3dpf to 4dpf, the liver size is smaller in *amd1^7-/-^* embryos (Aa1-a4). (B, C) Comparing the liver size in *amd1^7-/-^* embryos and control embryos using *fabp10* staining (Bb1-b6). The size of liver in *amd1^7-/-^* embryos was 62.4% (n=15, p<0.0001) , 61.0% (n=13, p<0.0001) and 46.3% (n=19, p<0.0001) of that in control embryos on 3dpf, 4dpf and 5dpf, respectively.(D, E) Comparing the liver size using *uox*staining (D). The size of liver in *amd1^7-/-^* embryos was 53.6% (n=12, p<0.01) ,45.5% (n=15, p<0.0001) and 46.0% (n=14, p<0.0001) of that in control embryos on 3dpf, 4dpf and 5dpf, respectively (E).(F, G) From 3dpf to 5dpf, in*Tg(fabp10:GFP)* transgenic embryos the data showed that the liver in *amd1^168-/-^* embryos was smaller than that in control embryos. (G) Statistical analysis for the liver size in *amd1^168-/-^* embryos with transgenic back groud and controls from 3dpf to 5dpf. In *amd1^168-/-^* embryos, the size of liver on 3dpf (0.58folds to control, n=13, p=< 0.0001), 4dpf (0.47folds to control, n=13, p=< 0.0001) and 5dpf (0.49folds to control, n=13, p=< 0.0001) is significantly decreased comparing with that in controls.(H, I) From 3dpf to 5dpf, *fabp10 in situ* staining showed that the liver in *amd1^168-/-^* embryos was smaller than that in control embryos. (I) Statistical analysis for the liver size in *amd1^168-/-^* embryos and controls from 3dpf to 5dpf. In *amd1^168-/-^* embryos, the size of liver on 3dpf (0.54folds to control, n=12, p=0.0004), 4dpf (0.59folds to control, n=10, p=< 0.0001) and 5dpf (0.45folds to control, n=11, p=< 0.0001) is significantly decreased comparing with that in controls. Values are reported as mean ± SEM. “**” P < 0.05,“***” P < 0.001, “****” P < 0.0001. Scale bars, 100μm.


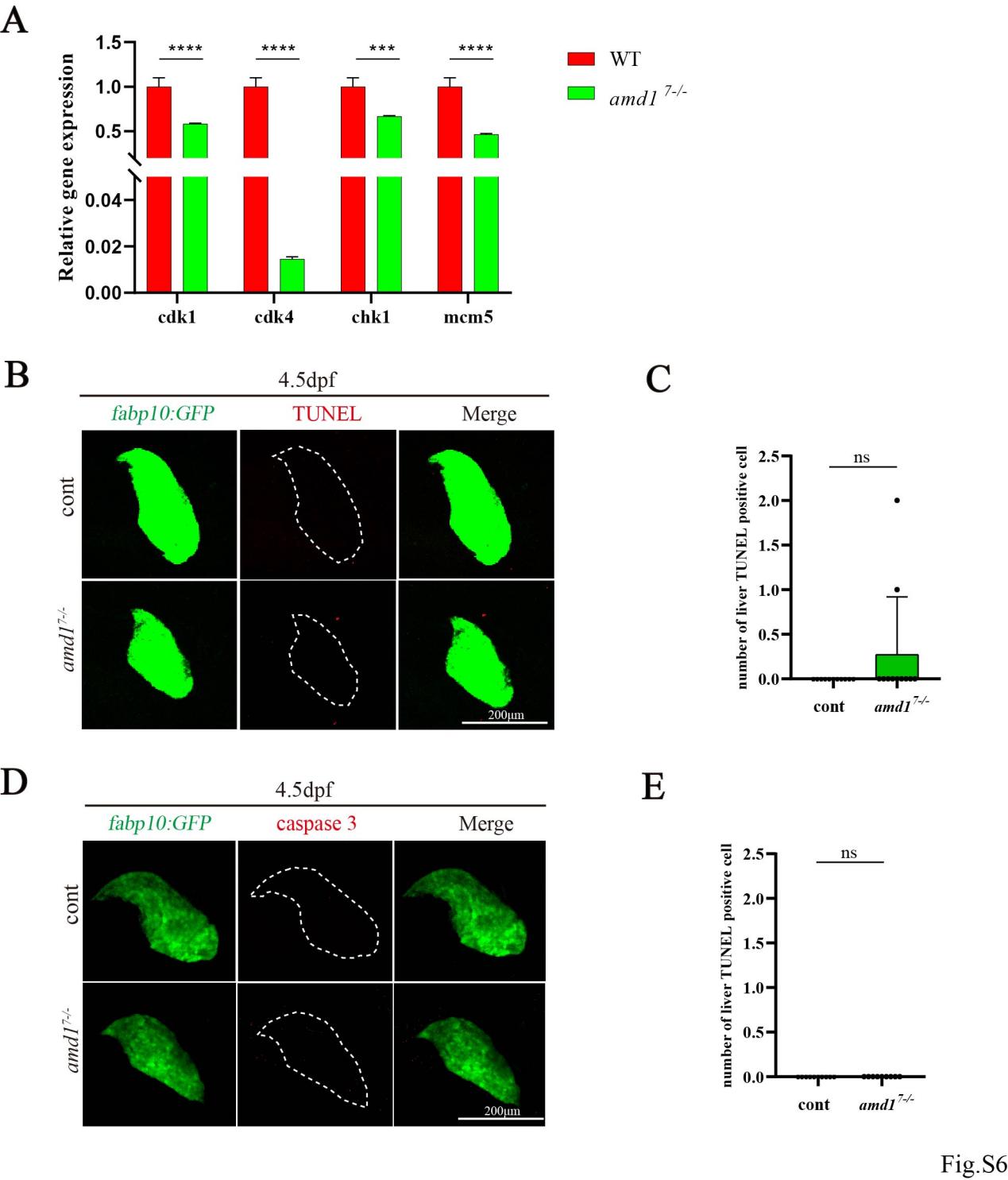


**Figure S6. Expression of cell cycle related genes and cell apoptosis examination in controls and *amd1* mutants**

(A) Expression of cell cycle related gene *cdk1*, *cdk4*, *chk1* and *mcm5*. Compared to wild type controls, the expression of *cdk1* (0.58 folds to control), *cdk4*(0.01 folds to control), *chk1*(0.67 folds to control) and *mcm5*(0.47 folds to control) were all downregulated. (B) Cell apoptosis was examined using TUNEL experiment. (C) Statistical analysis showed there is no significant difference between controls (n=11) and *amd1* mutants (n=11). (D-E) Cell apoptosis was examined using Caspase3 antibody staining (D). There is no difference between controls and *amd1* mutants (D, E). Values are reported as mean ± SEM. “***” P < 0.001, “****” P < 0.0001. Scale bars, 200μm.


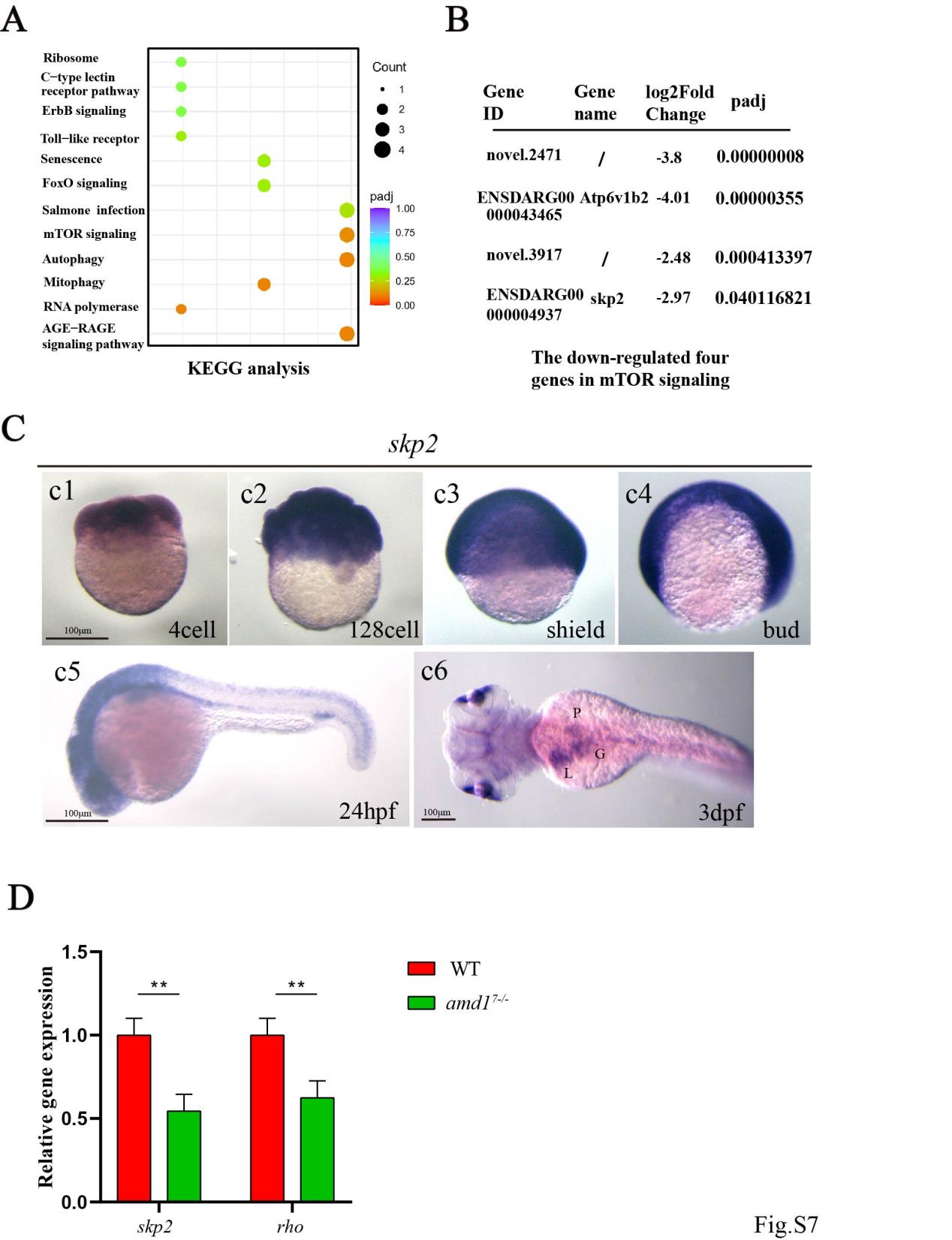


**Figure S7. *skp2* was downregulated in *amd1^7-/-^*embryos and the expression pattern**

(A-B) KEGG analysis showed that, in the down-regulated genes, some of them (four genes) were belonged to mTOR signaling (A). The down-regulated four genes in mTOR signaling were showed, among them *skp2* was significantly downregulated in *amd1^7-/-^* embryos (B). (C) The expression of *skp2* was examined at 4-cell stage (c1), 128-cell stage (c2), shield stage (c3), bud stage (c4), 24hpf (c5) and 3dpf (c6). On 3dpf, *skp2* was enriched in eyes, pancreas, liver and gut (c6). In c6, “L” means liver, “P” means pancreas, “G” menas gut. (D) RT-qPCR experiments showed that *skp2* (0.545 folds to control, p=0.0011) and *skp2* downstream gene *rho* (0.626 folds to control, p=0.0036) was downregulated in *amd1^7-/-^*embryos. Values are reported as mean ± SEM.“**” P < 0.01. Scale bars,100μm.


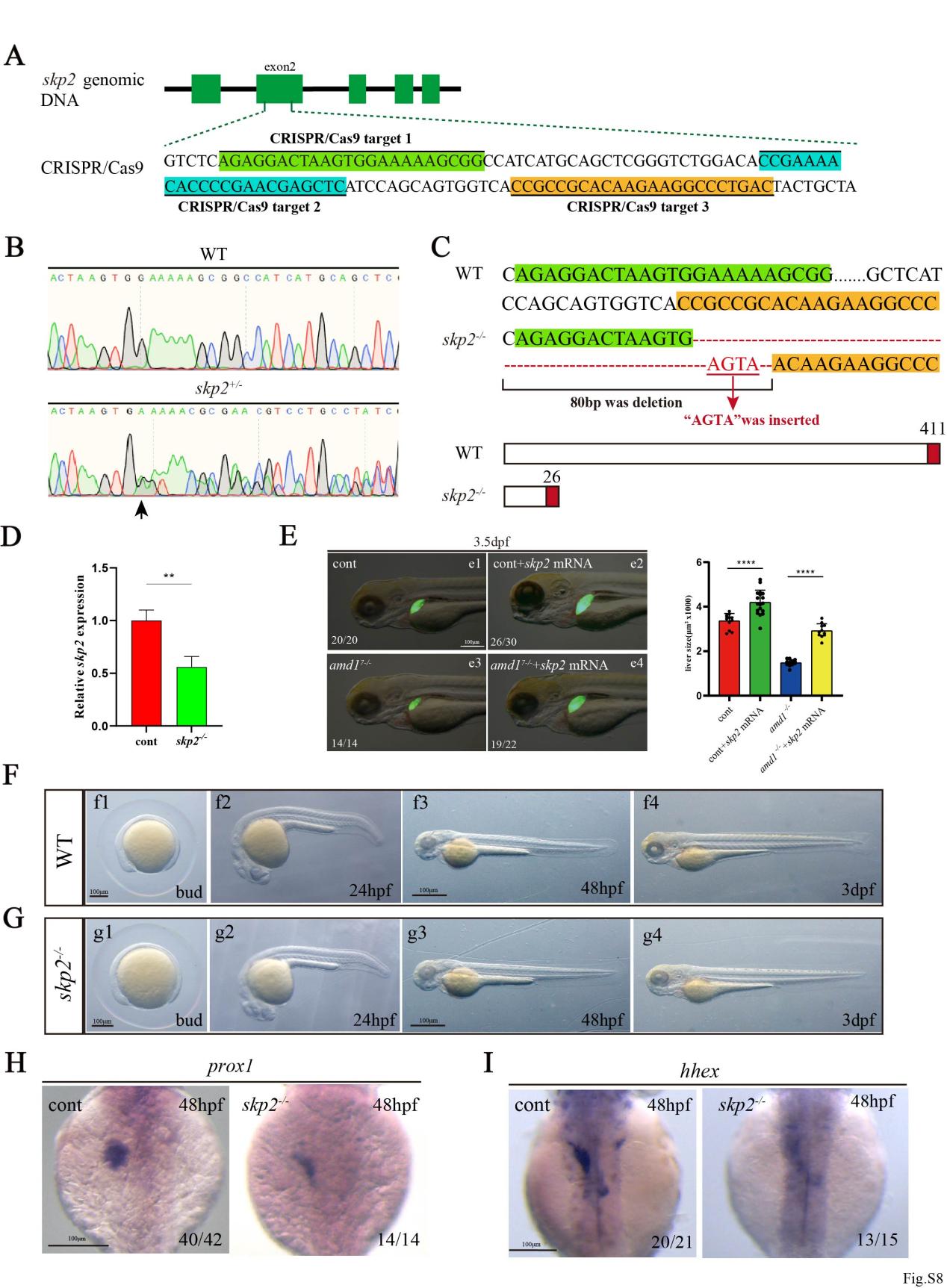


**Figure S8. *skp2* is required for liver development and lies downstream of *amd1***

(A) Three targets of *skp2* sgRNA in exon2 of *skp2* gene. (B) The sequencing results of *skp2* wild type (up sequence) and *skp2^+/-^*embryos (down sequence). (C) 80 nucleotides between “AAAAAGCGG” (green line labelled) and “ACAAGAAGG” (yellow line labelled) were deleted, but “AGTA” were added in these two sequences. This mutation gave rise to a frame-shift and produced a premature stop codon in the CDs region of *skp2*. The wild type Skp2 protein has 411 amino acids but mutated Skp2 protein only has 26 amino acids. (D) The expression of *skp2* mRNA in *skp2^-/-^*mutants was 55.2% of that in wild type controls on 4dpf. Values are reported as mean ± SEM. “**” P < 0.01. (E) *skp2* overexpression increased the size of liver. On 3.5 dpf, in wild type embryos injection of *skp2* mRNA increased the size of liver (86.6%, n=18); meanwhile the phenotype “smaller liver” in *amd1^7-/-^* embryos was rescued in 86.3% of embryos by injecting *skp2* mRNA (n=11).(F, G)The embryos of wild type at bud stage (f1), 24hpf (f2), 48hpf (f3) and 3dpf (f4)(F). (G) The skp2^-/-^ embryos at bud stage (g1), 24hpf (g2), 48hpf (g3) and 3dpf (g4). There is no distinct difference between wild type embryos and skp2^-/-^ embryos . (H) Expression of early liver marker *prox1* in embryos at 48hpf. The area of *prox1* expression is smaller in *skp2^-/-^* embryos than that in controls. (I) Expression of *hhex* in embryos at 48hpf. The area of *hhex* expression is smaller in *skp2^-/-^* embryos than that in controls. Scale bars, 100μm.“*” P < 0.05, “***” P < 0.001, “****” P < 0.0001, Scale bars, 100μm.


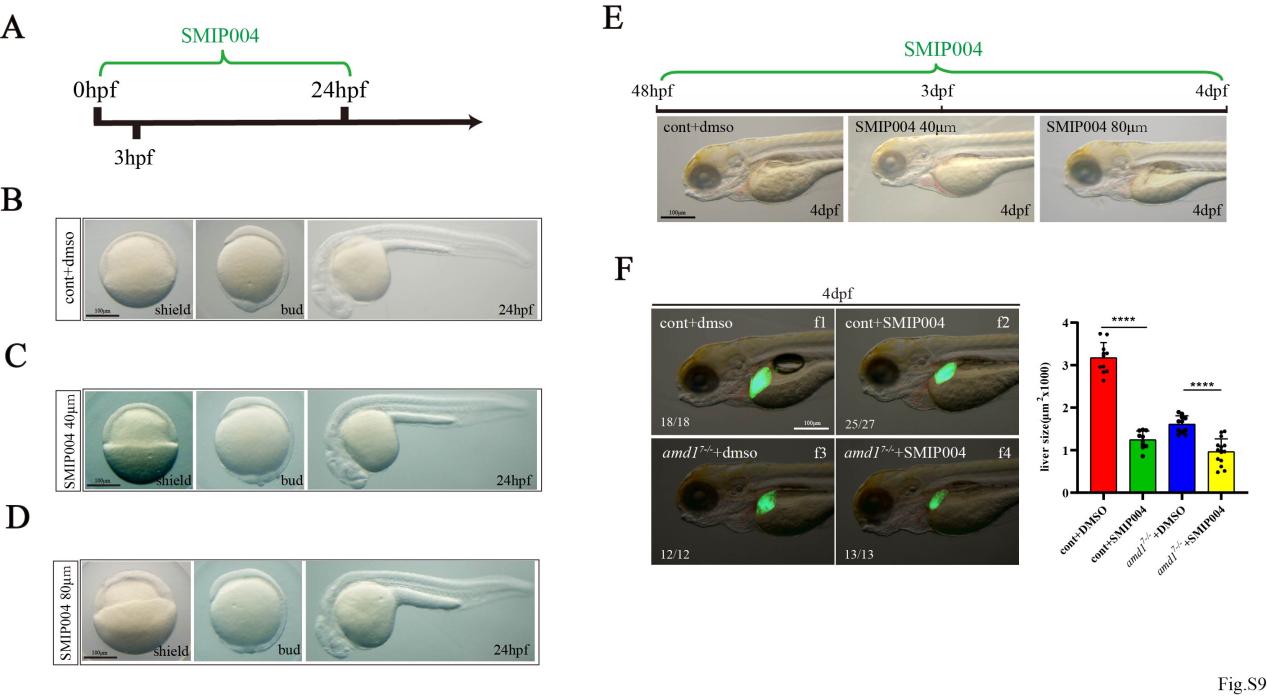


**Figure S9. *skp2* is required for liver growth and mediates *amd1* regulates liver development**

1. The schedule for SMIP004 treatment to embryos. (B-D) The overal embryonic phenotype after treating the embryos with SMIP004 at different concentration. (E) Treating the embryos with 80uM or 40uM SMIP004 from 48hpf to 4dpf did not lead to morphological defect on 4dpf. (F) After inhibiting skp2 activity, 92.6% of embryos (Ff2, n=10, p< 0.0001) displayed smaller liver than that in controls (Ff1, n=11); in amd1^7-/-^embryos, Skp2 inhibition (Ff4, 100%, n=13, p< 0.0001) made the liver much smaller that controls (Ff3, n=12).Values are reported as mean ± SEM. “*” P < 0.05, “***” P < 0.001, “****” P < 0.0001, Scale bars, 100μm.


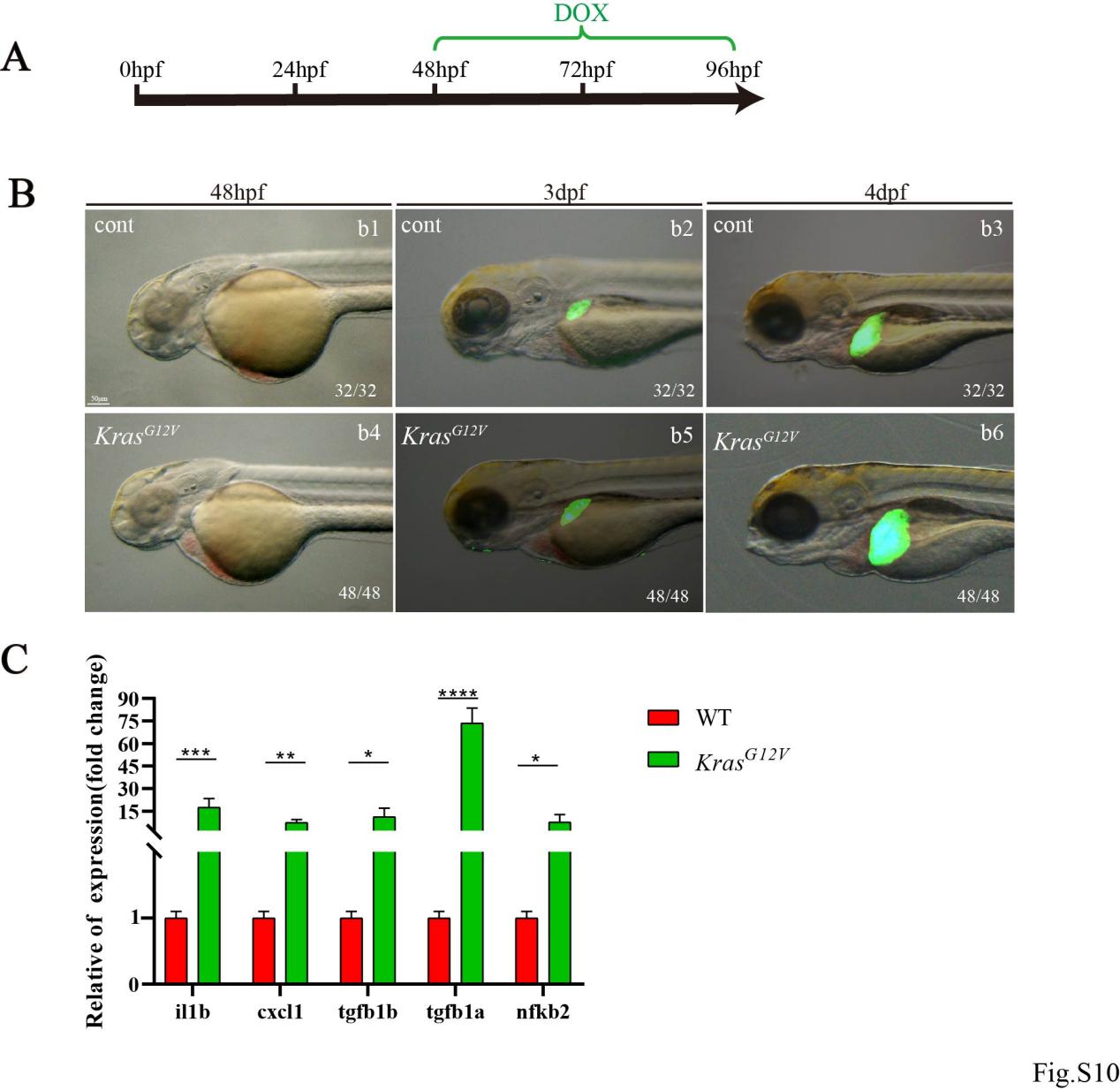


**Figure S10. Inducing HCC progress in zebrafish embryos**

(A) Schedule for using Dox to induce *Kras^G12V^* expression. Dox was used to treat embryos from 48hpf to stage required. (B) Comparing with the *Tg(fabp10:GFP)* embryos, inducing expression of *Kras^G12V^*from 48hpf resulted in larger liver on 3dpf (Bb5) and 4dpf (Bb6). (C) Comparing with controls, the expression of *il1b* (17.4 Folds, p=0.0003), *cxcl1* (7.3 Folds, p=0.0064), *tgfb1b* (11.0 Folds, p=0.0268), *tgfb1a* (73.6 Folds, p< 0.0001) and *nfkb2* (6.5 Folds, p=0.0439) was greatly upregulated in the hepatocytes in zebrafish HCC model. Values are reported as mean ± SEM. “*” P < 0.05, “**” P < 0.01, “***” P < 0.001, “*****” P < 0.0001. Scale bars, 50μm.
