# Supplementary material for: RNA-seq identifies Amd1 as a regulator of hepatocyte proliferation via Skp2 during liver development and hepatocellular carcinoma progression in zebrafish
Source: Genes Dis. 2024 Dec 11;12(5):101486. doi: 10.1016/j.gendis.2024.101486 (PMC12142500; doi:10.1016/j.gendis.2024.101486)
Supplement: Multimedia component 3 [file mmc3.docx]

Table S1

| Primer name | Check primer sequence(5′-3′) | |
| --- | --- | --- |
| skp2-sgRNA1-F | | taatacgactcactataGGCAGGGCCTTCTTGTGCGGgttttagagctagaa |
| skp2-sgRNA2-F  skp2-sgRNA3-F  sgRNA oligo2-R | | taatacgactcactataGGGCTCGTTCGGGGTGTTTTgttttagagctagaa  taatacgactcactataGGAGGACTAAGTGGAAAAAGgttttagagctagaa  AAAAGCACCGACTCGGTGCCACTTTTTCAAGTTGATAACGGACTA  GCCTTATTTTAACTTGCTATTTCTAGCTCTAAA AC |
| skp2-check-F | | CTGTAGGGTGTTTCAGGAGCTG |
| skp2-check-R  amd1-qpcr-F  amd1-qpcr-R  skp2-qpcr-F  skp2-qpcr-R  egfra-qpcr-F  egfra-qpcr-R  chmp4c-qpcr-F  chmp4c-qpcr-R  cyp17a1-qpcr-F  cyp17a1-qpcr-R  ptgis-qpcr-F  ptgis-qpcr-R  gatm-qpcr-F  gatm-qpcr-R  nos2b-qpcr-F  nos2b-qpcr-R  nos1-qpcr-F  nos1-qpcr-R  rho-qpcr-F  rho-qpcr-R  ahcy-qpcr-F  ahcy-qpcr-R  il1b-qpcr-F  il1b-qpcr-R  cxcl1-qpcr-F  cxcl1-qpcr-R  tgfb1b-qpcr-F  tgfb1b-qpcr-R  tgfb1a-qpcr-F  tgfb1a-qpcr-R  nfkb2-qpcr-F  nfkb2-qpcr-R | | GTCACAAACCTGATGACTCTCTTC  GGTTCAGTGATTGATGCCACG  CTCTGGTTCAGGGGTGATGTG  TTGTGCTCGCCAGACGGCC  CCCACCAGATCCACACTGT  CCATCTGCACCATTGATGTGTAC  CTTGGAGTCAGAAGGACTGGG  GATACGCCGCTAAAGCTATCAAAG  CAACTCGGCCAGTTCTGC  GAGCTGAACAAGTGGTCCTTTG  CTCAACAGGCGTCACCATC  GGACTGTTCAATCTTTGCTACAGC  GCTGCACTTGCAATTTGTTTCTCC  CACCATGGCGGACCAGC  CGCCGCATCCACTCGATG  GTTTCAAAAAGCAGCTTCTCAGCC  GCCCTTGAACATTAAACTCCTTGC  CAAATACGCCACCAACAAAGG  GCTTTCCATCCTTGCTGCATAC  GCCATGAACGGTACAGAGG  CGCAGCTTCTTGTGCTCG  GCTGGCTGTTTGCACATG  CAGTCTCTCCCTTCCAGGC  GCCTGTGTGTTTGGGAATCT  TGATAAACCAACCGGGACAT  GGCATTCACACCCAAAGCG  GCGAGCACGATTCACGAGAG  AGCCCGAGTCTCAGAACA  CATCAGCAGGATGTGACATC  AGCAGAATTGCGTCTTCGGA  TCAAATGAGAGCCAGCGGTT  GGAGTGGTAGAAGTCTTAACG  TTTTGCCTAACTCTTTGG |
